# Simulating far infrared spectra of Zn_1-x_Mn_x_Se/GaAs epifilms, MnSe/ZnSe superlattices and predicting impurity modes of N, P defects in Zn_1-x_Mn_x_Se

**DOI:** 10.1080/14686996.2016.1222495

**Published:** 2016-12-21

**Authors:** Devki N. Talwar, Tzuen-Rong Yang, Wu-Ching Chou

**Affiliations:** ^a^ Department of Physics, Indiana University of Pennsylvania, Indiana, PA, USA; ^b^ Department of Physics, National Taiwan Normal University, Taipei, Taiwan, ROC; ^c^ Department of Electrophysics, National Chio Tung University, Hsinchu, Taiwan, ROC

**Keywords:** ZnMnSe/GaAs semiconductors, infrared spectra, MnSe/ZnSe superlattices, impurity modes, ATM-Greens function theory, 10 Engineering and Structural materials, 201 Electronics / Semiconductor / TCOs, 105 Low-Dimension (1D/2D) materials, 204 Optics / Optical applications, 505 Optical / Molecular spectroscopy

## Abstract

A comprehensive lattice dynamical study is reported to emphasize the vibrational behavior of perfect/imperfect zinc-blende (zb) ZnSe, MnSe and Zn_1−x_Mn_x_Se alloys. Low temperature far-infrared (FIR) reflectivity measurements performed on a series of molecular beam epitaxy grown Zn_1−x_Mn_x_Se/GaAs (001) epilayers have a typical ‘intermediate-phonon-mode’ behavior. Besides perceiving ZnSe- and MnSe-like TO-phonon resonances, the study also revealed a weak Mn alloy-disorder mode below MnSe band. A classical effective-medium theory of multilayer optics is used to evaluate dielectric tensors of both epilayers and substrate for simulating reflectivity and transmission spectra of ultrathin epifilms and superlattices at near normal and/or oblique incidence. In the framework of a realistic rigid-ion model and exploiting an average *t*-matrix Greens function (ATM-GF) theory we appraised the vibrational properties of nitrogen and phosphorous doped Zn-Mn chalcogenides. Lattice relaxations around isolated N_Se_ (P_Se_) defects in ZnSe and zb MnSe are evaluated by first principles bond-orbital model that helped construct perturbation models for simulating the localized vibrational modes (LVMs). Calculated shift of impurity modes for isotopic ^14^N_Se_ (^15^N_Se_) defects in ZnSe offered a strong revelation of an inflexible defect–host interaction. By retaining force constant change parameter of ^14^N_Se_ (^15^N_Se_) in heavily N-doped ZnSe, the ATM-GF theory predicted (a) three non-degenerate LVMs for the photoluminescence defect center ^V^_Se_-Zn-^14^N_Se_ (^V^_Se_-Zn-^15^N_Se_) of *C*_s_ symmetry, and (b) six impurity modes for the second nearest-neighbor N_Se_-Zn-N_Se_ pair defect of *C*_2v_ symmetry. From the range of simulated defect modes, we have ruled out the possibility of N-pairs and justified the presence of V_Se_-Zn-N_Se_ complex centers – likely to be responsible for the observed large absorption bandwidth in highly N-doped ZnSe. High resolution measurements of FIR absorption and/or Raman scattering spectroscopy are needed to validate the accuracy of our theoretical conjectures.

## Graphical abstract

**Figure:**
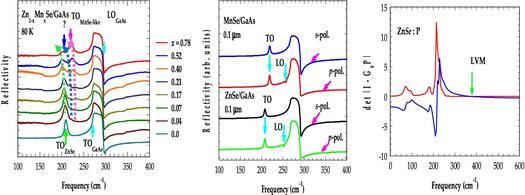

## 1. Introduction

Spin-related phenomenon in electronics (spintronics) has recently emerged as an interdisciplinary field of nanoscience whose aim is to use charge and spin-dependent properties to realize solid-state devices with more advanced capabilities than semiconductor-based integrated circuits (SBICs) and/or magnetic memory-based chips (MMBCs).[1–8] The SBICs used in digital electronics, analog circuits, and optoelectronics are exploited to control the flow of charge carriers (electrons or holes) in semiconductors, thin films and/or hetero-structures to create efficient devices with small and precisely delineated dimensions. In MMBC technology, however, the spin of electrons is the key parameter responsible for the magnetic moment. Earlier discoveries [9–12] of the carrier-induced ferromagnetism in Mn-based zinc-blende (zb) III–V compounds followed by the predictions and observations of ferromagnetism in p-type II–VI materials [13] have inspired researchers to examine the physics of unexplored combinations of hetero-structures and ferromagnetic semiconductors. The contemporary research on spintronics has implicated both the magnetic materials and the structures of hybrid ferromagnetic compounds – especially the Mn-based ternary alloys of the II-VI zb family of diluted magnetic semiconductors (DMSs): namely
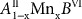


where *A* ^II^ = Be, Mg, Zn, Cd, Hg and *B* ^VI^ = O, S, Se, Te. The *sp* ^3^- *d* exchange interactions between Mn spins and charge carriers produced localized magnetic moments, making DMS materials quite promising for engineering novel electronic devices including spin injectors, magneto-optical switches, aligners, filters, sensors and gamma-ray detectors [14–19]. In 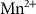 alloys, as *x* increases the *A* ^II^ cations are likely to replace 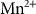 ions – impacting their degrees of hybridization as well as electrical, optical, structural and vibrational properties.[20–27]

These novel characteristics have motivated many studies of the electrical properties of zb Zn_1-x_Mn_x_Se (for *x* ≤ 0.35) alloys prepared either by Bridgman method [14–18] and/or ultrathin epilayers conceived by molecular beam epitaxy (MBE), metalorganic chemical vapor deposition (MOCVD), pulsed laser evaporation and epitaxy (PLEE), atomic layer epitaxy (ALE) and radio frequency (RF) sputtering techniques.[19–27] A large atomic radius of Mn (1.79 Å) compared to Zn (1.53 Å) caused difficulties incorporating higher amounts of 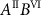 into 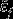 compounds. Successful efforts have been made, however, in recent years to grow by MBE the better quality of zb Zn_1-x_Mn_x_Se epilayers (*x* ≤ 0.78) [20] and (MnSe)_m_/(ZnSe)_n_ superlattices (SLs) on (001) GaAs substrates [28–32] to explore their use for engineering spin-based electronic devices.[14–19] These novel materials will not be of much practical use if they cannot be doped. Many efforts to fabricate p-n junctions (indispensable for commercial devices) based on II–VI materials were hampered earlier by doping impediments. Despite several decades of extensive research, it is still not very clear why some of the wide-band-gap materials including ZnSe can be made n-type but not p-type while others can be doped p-type but not n-type.[31,32] The mechanism of such a predicament has still remained a mystery. At low doping levels both nitrogen (N) and phosphorous (P) impurities in ZnSe are believed to occupy the Se-sites (N_Se_, P_Se_), forming shallow acceptor-like states.[33] However, heavily doped N and P impurities (>10^18^ cm^−3^) in ZnSe are highly compensated by native defects and anticipated to form deep acceptor-like states. While the nature of compensating centers in p-ZnMnSe still remained largely unknown – there are speculations, however, that self-compensation in heavily N doped ZnSe arise either from a donor-like complex (e.g. V_Se_-Zn-N_Se_), N-pairs (N_Se_-N_Se_), N split interstitials on Se-sites (N_int_-N_int_)_Se_, and/or anti-site pairs (N_Zn_-N_Se_).[33–38] It is also suggested that in heavily N doped ZnSe a cluster of N_Se_ such as (N_Se_)_n_-Zn could have played a role as a deep acceptor.[33] As the masses of N and P are much less than the mass of Se in ZnSe, MnSe and/or dilute Zn_1-x_Mn_x_Se alloys, the use of local vibrational mode (LVM) spectroscopy blended with a realistic lattice dynamical average-*t*-matrix Green’s function (ATM-GF) theory could be indispensable for providing opportunities to examine (a) the shifts of LVMs of isotopic species (e.g. ^14^N_Se_ and ^15^N_Se_), and (b) identifying the site selectivity of isolated (e.g. P_Se_ and N_Se_) and complex defect centers [e.g. Mn_Zn_-P_Se_ (*C*_3v_), V_Se_-Zn-N_Se_ (*C*_s_), and V_Se_-Mn_Zn_-N_Se_ (*C*_s_)] of expected symmetries.

Far infrared (FIR) reflectivity and/or Raman scattering (RS) spectroscopies [20–28] have proven to be quite valuable for material characterizations, i.e. assessing the information about lattice phonons, site selectivity of intrinsic and/or doped defects, strain, phase transition, disorder and alloy compositions. Earlier testimonies by FIR reflectivity [26–28] at near normal incidence for the long-wavelength optical phonons in zb Zn_1-x_Mn_x_Se/GaAs (001) epilayers (x ≤ 0.43) have ascertained a typical ‘intermediate-mode behavior’ – revealing strong evidence of the ZnSe-like optical phonons and only a partial insinuation of the MnSe-like modes. It is to be noted that in RS spectroscopy [22,23] the TO mode is not allowed for the epitaxially grown zb epifilms with [001] growth axis in the backscattering geometry. This result is quite significant because a large number of zb Zn_1-x_Mn_x_Se epifilms, multi-quantum wells (MQWs) and (MnSe)_m_/(ZnSe)_n_ SLs are prepared having the [001] growth axis. In such cases one needs an alternative method to perceive the optical (LO and TO) modes. The optical phonons can be observed in ultrathin zb epifilms irrespective of their orientations by resorting to FIR transmission and/or reflection [39] at an oblique incidence (Berreman’s [40] effect). While limited efforts are made by IR/RS spectroscopy to probe LVMs of isolated N and P defects in ZnSe at low doping levels, no systematic strives exist for exploring the impurity modes at high doping levels especially appraising the nature of self-compensating ‘impurity-defect’ complexes. For quantitative analyses of the dynamical properties, a complete knowledge of phonons [41,42] for ZnSe and zb MnSe is crucial per se to help simulate the lattice dynamics of perfect/imperfect materials.

**Figure 1.**
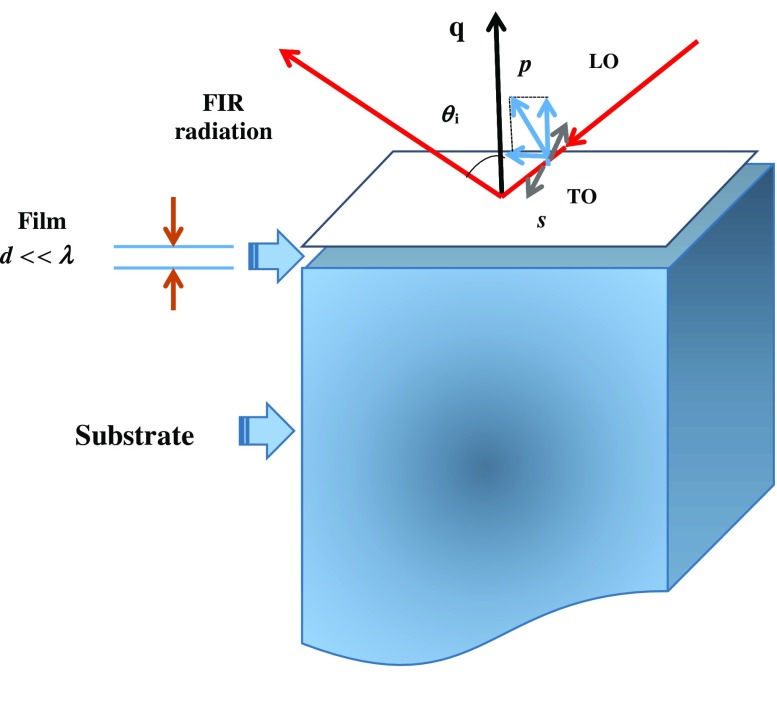
A polar semiconductor thin film of thickness *d* on a thick substrate. The directions of *s* (perpendicular, ⊥) and *p* (parallel, ||) components of the FIR radiation incident are at an oblique angle (*θ*_i_) to the surface of a film (perpendicular to the phonon wave-vector 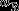) of thickness *d* << *λ* grown on a thick substrate.

In this study we emphasize the dynamical behavior of N_Se_ and P_Se_ defects in binary ZnSe, zb MnSe and ternary Zn_1−x_Mn_x_Se alloys. We have used a high resolution infrared spectrometer (Bruker IFS 120) to acquire low temperature (80 K) FIR reflectivity spectra of various MBE grown (cf. Sections 2.1–2.2) Zn_1-x_Mn_x_Se/GaAs (001) epilayers with *x* ≤ 0.78. The rationale behind such experiments is to simulate the FIR reflectivity spectra of Zn_1-x_Mn_x_Se epifilms and assess the validity of a classical dielectric response theory (cf. Sections 3.1–3.4). The traditional multilayer optics within a three-phase (ambient/film/substrate) model (see Figure 1) is adopted here to calculate thickness dependent reflectance and transmission spectra of ultrathin Zn_1-x_Mn_x_Se epilayers and (MnSe)_m_/(ZnSe)_n_ SLs at oblique incidence. In the doped samples, the effects of lattice phonons and charge carriers are incorporated with contributions from Drude and Lorentzian harmonic oscillators to model dielectric functions of epifilm 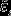
and substrate 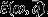. In Section 3.5 we present a succinct description of macroscopic lattice dynamical theory within the ATM-GF formalism for investigating the LVMs [43–45] of N_Se_ and P_Se_ defects in ZnSe, zb MnSe and ZnMnSe. Based on the classical dielectric response theory our simulated results of reflectivity and transmission spectra for ternary Zn_1-x_Mn_x_Se/GaAs (001) epifilms and (MnSe)_m_/(ZnSe)_n_ SLs compared favorably well with the experimental data (cf. Sections 4.1–4.4). The rigid-ion model (RIM) calculation of phonon dispersions for ZnSe and zb MnSe is shown to possess the required accuracies when compared with the existing inelastic neutron scattering (ZnSe) [46–48] and first principles [49] data. Earlier, the ATM-GF theory of imperfect materials (cf. Sections 4.5–4.6) offered a clear delineation of chemical trends attaining simple physical understanding of the bonding mechanism through magnitudes of impurity-host interactions.[50] Here, we reaffirm that at low doping levels the ATM-GF approach is quite adequate for elucidating the observed isotopic shifts of N_Se_ local modes in ZnSe. At higher N- (P-) doping level, the method has the potential of establishing the microstructures involving N- (P-) impurities and intrinsic defects in ZnSe. Our simulation of LVMs for ^14^N_Se_ (^15^N_Se_), P_Se_ defects in ZnSe (*T*_d_ symmetry) has offered (cf. Section 4.6a) triply degenerate *F*_2_ modes near 550 cm^−1^ (535 cm^−1^), 374 cm^−1^, respectively in excellent agreement with the IR [51] and Raman scattering [52] data. In zb MnSe, we predict *F*_2_ local modes of ^14^N_Se_ (^15^N_Se_), P_Se_ near 518 cm^−1^ (503 cm^−1^), 358 cm^−1^, respectively. In ^14^ N- (^15^ N-) and P-doped Zn_1-x_Mn_x_Se with low Mn composition, one would expect the formation of nearest neighbor (NN) Mn_Zn_–^14^ N_Se_ (Mn_Zn_–^15^ N_Se_), Mn_Zn_–P_Se_ pairs of *C*_3v_ symmetry to cause (cf. Section 4.6b) the splitting of ^14^N (^15^N), P_Se_ LVMs. In heavily N-doped ZnSe the origin of a strong charge compensation is construed by partial passivation of acceptors with formation of self-compensated second nearest neighbor (2nd NN) donor-like complex centers V_Se_-N_Se_ of *C*_s_ symmetry (cf. Section 4.6c). The simulated results of reflectivity and transmission spectra for Zn_1-x_Mn_x_Se epilayers, (MnSe)_m_/(ZnSe)_n_ SLs and the LVMs of N- and P- in Zn_1-x_Mn_x_Se are compared and discussed with the experimental data and a summary of concluding remarks presented in Section 5.

## 2. Experimental

### 2.1. Growth of Zn_1-x_Mn_x_Se/GaAs (001) epilayers

The Zn_1-x_Mn_x_Se epilayers used in this study with different Mn-compositions (*x* ≤ 0.78) were grown on GaAs (001) substrate by exploiting a Veeco Applied EPI 620 MBE system. The EPI 40 cc low temperature cells were employed for evaporating the elemental solid sources of Zn and Se while a standard temperature EPI 40 cc cell was used for the evaporation of Mn solid source. Each cell had its own shutter to control the growth time. There was a main shutter between the sources and the substrate for protecting it from evaporating before growth. Although the cell temperature of Se was kept at 180°C, the cell temperatures of Zn and Mn were varied, however, from 250°C to 300°C and from 700°C to 750°C, respectively. Different substrate temperatures between 280°C and 340°C were used during the growth of Zn_1-x_Mn_x_Se epilayers with *x* ≤ 0.78. By setting the growth rate at about 0.3–0.4 μm h^–1^, various samples were prepared having epilayer thickness ranging between ~0.1 and 4 μm. Again, we employed the reflection high energy electron diffraction (RHEED) method for accurately monitoring the crystal structure of Zn_1-x_Mn_x_Se during the growth process. The Mn composition in each epilayer was determined by applying energy dispersive X-ray (EDX) methodology. All the MBE grown Zn_1-x_Mn_x_Se/GaAs (001) samples were found to have the zb structure in agreement with the results published earlier.[20]

### 2.2 Far infrared reflectance

**Figure 2.**
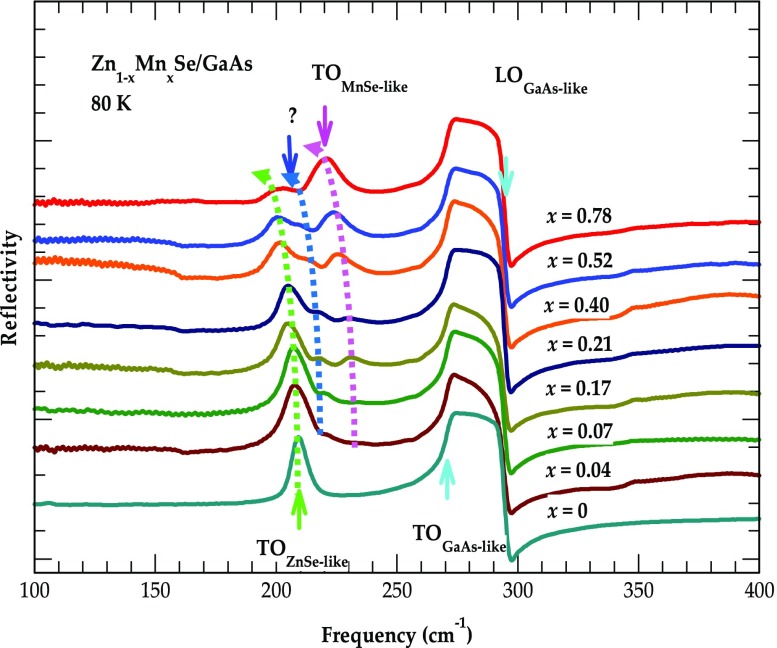
Low temperature FIR reflectivity spectra (80 K) for eight different MBE grown Zn_1-x_Mn_x_Se/GaAs (001) epilayers with Mn composition varying between 0 and 0.78. The spectrum of each sample is shifted upwards on the reflectivity scale for clarity. In ZnSe/GaAs (001) the spectra show a sharp ZnSe-like TO mode and GaAs TO, LO modes in the reststrahlen region. In Zn_1-x_Mn_x_Se/GaAs (001) the reflectivity spectra show two additional phonon modes between ZnSe-like (green-dotted line) and GaAs-like bands. Besides perceiving a zb MnSe-like TO mode (magenta-dotted line) we find a weak trait (blue-dotted line) below the MnSe-TO mode, believed to have its link to Mn alloy disorder (see text).

By using a high resolution Bruker IFS 120 Fourier transform IR (FTIR) spectrometer (Bruker Optics Inc., Billercia, MA, USA), we have performed the low temperature (80 K) reflectivity measurements at near normal incidence (*θ*_i_ ≈ 8^o^) on various Zn_1- x_Mn_x_Se/GaAs (001) samples over the frequency range of ~ 60 to 5000 cm^−1^. For reflectivity study in the FIR region (60–700 cm^−1^) a mercury lamp was used in combination with helium cooled Si bolometer, while in the mid infrared (MIR) region (700–5000 cm^−1^) we employed a mercury cadmium telluride (MCT)/KBr detector, a KBr beam splitter along with a globar light source. In the reflectivity measurements we exploited a gold (Au) mirror – whose absolute reflectance is measured directly as a reference to specify the IR spectra of Zn_1-x_Mn_x_Se. In Figure 2, the results of FIR spectra are displayed for eight MBE grown Zn_1-x_Mn_x_Se/GaAs (001) samples with different Mn compositions *x*, ranging from 0 to 0.78. The rationale behind such experiments is to simulate FIR spectra of Zn_1-x_Mn_x_Se and to assess the validity of a classical model described in Sections 3.1–3.4.

Figure 2 identifies ZnSe-like and MnSe-like transverse optical phonon frequencies near ~208.5 cm^−1^ (green arrow:*ω*_*TO*1_) and ~219.3 cm^−1^ (magenta arrow:*ω*_*TO*2_) for *x* = 0 and *x* = 0.78, respectively. All the *ω*_*TOj*_ resonance modes are shown temperature dependence variations. The ZnSe-like (MnSe-like) TO phonons unveiled ~3–4 cm^−1^ (~5 cm^−1^) blue shift when the temperature is decreased from 300 K to 80 K. Besides TO phonons of ZnSe and (zb-MnSe), we also noticed a weak trait (dark blue arrow) just below the MnSe-like TO band. In Zn_1-x_Mn_x_Se alloys, the composition-dependent optical modes *ω*_*LOj*_ and *ω*_*TOj*_ are related to their respective oscillator strengths. In Table 1 we have recorded appropriate values of ZnSe- and zb MnSe-like optical phonons along with mode broadening parameters extracted from theoretical fits to the experimental (IR reflectivity) spectra.

**Table 1.** Composition-dependent ZnSe-like [MnSe-like] optical phonon modes *ω*_*TO*_, *ω*_*LO*_ [*ω*_*TO*_, *ω* ^*I*^_*LO*_] and their respective broadening Γ_*TO*_, Γ_*LO*_ [Γ_*TO*_, Γ^I^_*LO*_] parameters (see text) extracted from theoretical fits to the experimental FIR reflectivity (80 K) spectra (see Figure 2) of eight MBE grown Zn_1−x_Mn_x_Se/GaAs (001) epilayers using the classical dielectric response theory [Equation (3)].

ZnSe-like						MnSe-like			
Sample	*x*	(cm^–1^)	(cm^–1^)	(cm^–1^)	(cm^–1^)	*ω*_*TO*_ (cm^–1^)	*ω*^*I*^_*LO*_ (cm^–1^)	(cm^–1^)	(cm^–1^)
1	0	208.0	254.0	1.7	1.7				
2	0.04	207.0	254.3	1.92	1.92				
3	0.07	206.4	255.4	2.31	2.37	228.3	227.9	7.23	7.19
4	0.17	206.1	256.2	4.07	4.13	226.4	221.7	9.32	7.27
5	0.21	205.7	257.3	4.32	4.10	224.9	216.5	9.91	7.70
6	0.40	202.8	257.9	5.02	5.3	221.1	214.1	10.41	7.96
7	0.52	200.5	258.6	7.21	3.40	219.6	211.4	10.89	7.12
8	0.78	199.7	259.0	7.8	3.7	218.7	210.7	8.10	9.75

## 3. Theoretical

From an experimental standpoint, the information about the lattice dynamics of perfect and/or imperfect semiconductors can be achieved by using optical spectroscopy – i.e. measuring the frequency-dependent response of the material to an external probe. Inelastic neutron scattering – the most efficient method for revealing phonon dispersions in bulk (e.g. ZnS, ZnSe, ZnTe and CdTe) semiconductors [46–48] has not yet been applied to the zb phase of Mn-chalcogenides (MnS, MnSe, MnTe). While many optical measurements [21–28] do exist in Zn_1-x_Mn_x_Se from IR, RS and spectroscopic ellipsometry – the expositions of such studies, however, differ significantly. Accurate analysis of the optical data for Zn_1-x_Mn_x_Se epilayers and (MnSe)_m_/(ZnSe)_n_ SLs along with the assessment of dynamical characteristics for p-type dopants (e.g. N_Se_, P_Se_) in Zn_1-x_Mn_x_Se is indispensable for comprehending their structural and vibrational properties.

### 3.1. Classical theory of FIR reflectivity for binary compounds

The physical process described by the interactions between electromagnetic radiation and crystal lattices in the FIR region can be articulated by frequency and wave-vector dependent dielectric response function 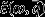 In the zb type polar semiconductors, there are two main processes that contribute to 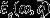: (a) the free-charge carrier effect [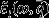] in n- or p-type doped materials – caused by electrons in the conduction band or holes in the valence band, and (b) the lattice effect [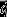] from optical phonons. In the limiting case, where the wave-vector 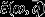 approaches zero, the general form of dielectric function 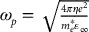 for doped materials developed by Drude, Lorentz and others takes the classical form [53]:


(1) 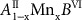



with(1a) 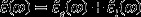

(1b) 
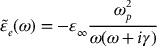



where the term 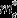 in Equation (1a) represents the plasma frequency; *η* stands for the free-carrier density; 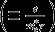 the effective mass; *e* the electronic charge; *γ* (Γ) signifies plasmon (phonon) damping coefficient; *S* in Equation (1b) implies the oscillator strength; and *μ* 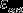 is the mobility of free charge carriers. Clearly, the simulation of dielectric function [Equation (1)] is the foundation for relating the phonon frequencies to the FIR reflectivity spectra and appraising the plasma frequency in semiconductor materials.

### 3.2. Ternary alloys

In polar alloy semiconductors (e.g. *A*_1-*x*_ *B*_*x*_ *C*) – the complex dielectric function [Equation (1)] can be re-written as:(2) 
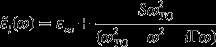



where the term 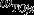 represents high-frequency dielectric constant taken as a weighted average between the corresponding values of pure binary compounds *AC*-*BC*; *S*_*jx*_ is the composition dependent oscillator strength; 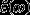
and Γ_*jx*_ represent, respectively, the resonance frequency and damping parameters of the *j*th TO phonon for different *x*.

In Zn_1-x_Mn_x_Se alloys with multiple phonon modes having large TO-LO splitting and anharmonicity, the model presented in Equation (2) is found inadequate for correctly defining 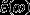 and thus portraying the reflectivity spectra. In the mixed alloys, we have thus evaluated the phonon contributions to 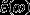 by using the product of individual oscillator terms, i.e.:(3) 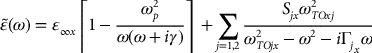



and for the doped materials the contributions of free charge carriers to 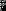 is appropriately included using Equation (1a). Once the dielectric function of binary and/or ternary alloy is known – the reflectance coefficient 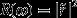 at near normal incidence:


(4) 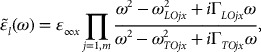



can be easily simulated and, hence, the power reflection 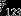.

### 3.3. Reflection/transmission of epifilms at near normal incidence

To assess the FIR reflectance/transmission spectra for epifilms of thickness *d* grown on a given substrate (cf. Figure 1), we have used a standard methodology of multilayer optics by integrating a three phase (ambient/film/substrate) model. At near normal incidence, the amplitude of reflection coefficient 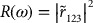 or reflectivity 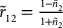 can be derived in a straightforward manner by using [53]:


(5) 
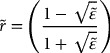



where 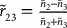; 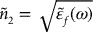 with 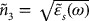 and 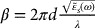 are the Fresnel coefficients; the term 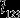 in Equation (5) represents the phase multiplier and *λ* is the wavelength. An expression similar to Equation (5) for the transmission coefficient 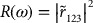 at near normal incidence can be derived.[53] The articulation of simulating reflection 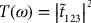 (transmission 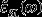) at an oblique incidence (Berreman’s effect [40]) is, however, a little more involved.

For AC/BC SLs (with A = Mn, B = Zn and C = Se), the above approach of calculating reflectivity/transmission spectra of thin films can be easily expanded. In the framework of an effective medium theory, the dielectric function of SL [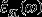] is a diagonal tensor with components expressed as the weighted averages of dielectric functions of constituent layers. For radiation having electric field parallel to the SL layers, the 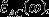 can be expressed as:(6) 
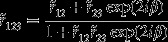



where 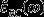 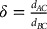 are, respectively, the dielectric functions of the two alternating layers of materials *AC*, *BC* forming SL, and 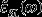 is the ratio of their thicknesses. For radiation incident at an oblique angle the above Equations (5) and (6) become complicated [53] functions (cf. Section 3.4) of *θ*_*i*_ and polarization (*s*-, *p*-).

### 3.4. FIR reflection and transmission at oblique incidence

Following Piro,[54] the analytical expressions of *R*(*ω*) [*T*(*ω*)] are derived for a SL at an arbitrary angle of incidence *θ*_*i*_. The SL consisting of two *AC*, *BC* layers grown alternately on a substrate are treated as an isotropic medium. The dielectric tensor of SL 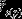 with uniaxial crystal structure having *D*_*2d*_ symmetry takes the form:


(7) 
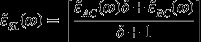



Here, the *z*-axis is set along the optical axis perpendicular to the plane of the SL. If the wavelength *λ* of incident radiation is large compared to the SL period *d* (=*d*_*AC*_ + *d*_*BC*_), one can use boundary conditions to write:(8a) 
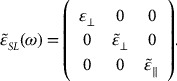



and(8b) 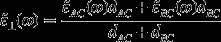. 


The amplitude reflectance 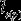 (transmittance 
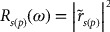
) and power reflectance 
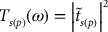
 (transmittance 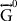) can be obtained using:(9a) 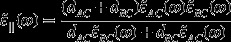

(9b) 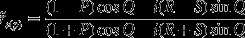



where the terms *P*, *Q*, *R*, *S* and *ξ*, *ζ* in the *s*(*p*)- polarization are given elsewhere.[53–55]

### 3.5. Impurity vibrational modes

For extracting impurity–host interactions from the spectroscopic data on LVMs, two equally reliable theoretical schemes have been attempted: (a) a *microscopic* analysis based on the density functional theory (DFT),[56,57] and (b) a *macroscopic* approach [50] based on the general treatment of lattice dynamics within the Green’s function theory.[41–45] For isoelectronic defects, the former method [56,57] requires heavy computation and it is much more cumbersome for non-isoelectronic (charged) impurities and their complexes. The advantage of adopting the second approach [50] over the first one is that it allows the coupling of vibrations of defect centers to the bulk materials. Except for the LVMs of oxygen doped CdTe,[56,57] no microscopic calculations are known for the vibrational modes of ‘impurity-defect’ complexes involving either N and/or P in ZnSe and/or zb MnSe.

For simulating the dynamical behavior of N_Se_ and P_Se_ acceptors in Zn_1-x_Mn_x_Se by the *macroscopic* method one needs to evaluate the lattice Green’s function 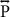 and perturbation 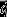 matrices by assimilating the host lattice phonons (eigenvalues and eigenvectors) from a reliable lattice dynamical scheme. By exploiting an optimized RIM,[58] we have generated phonons at 64,000 wave vector 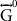 points in the Brillouin zone. Its accuracy is tested by comparing the phonon dispersions with inelastic neutron scattering [46–48] measurements and first principles [49] calculations (cf. Section 4). Other physical quantities acquired [e.g. *C*_v_(T), *Θ*_*D*_(T)] a posteriori for the perfect crystals [59] achieving good agreement with the experimental data offered additional support to the reliability of RIM. The Green’s function matrix 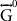
elements of ZnSe and zb MnSe are evaluated numerically following traditional procedures – first obtaining the imaginary part of 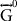 by using phonons at a sample of wave vectors in the reduced Brillouin zone and then assessing the real part of 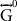
via links provided by the Kramers–Krönig relations.

Besides simulating 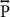 the other important issue is to give an adequate representation to the impurity perturbations 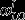. The elements of perturbation matrices are appropriately constructed by including mass change at the impurity sites and the effects of lattice relaxation to account for the impurity–host interactions. In calculating the LVMs of various isolated and complex defect centers, we took advantage of the symmetry-adapted algorithm to help compare theoretical results with the spectroscopic data. The frequencies of impurity vibrational modes in different irreducible representations (*μ*Γ) of the point group symmetries are obtained by solving the equation:[45](10) 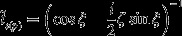



The zeros of Equation (10) give poles at energies either above the maximum phonon frequency *ω*_m_ of the host lattice (LVMs) or within the lattice bands (band modes) where the density of phonon states is low.

## 4. Numerical calculations results and discussions

### 4.1. Reflectivity spectra of Zn_1-x_Mn_x_Se/GaAs (001)

In Figure 2, we have displayed our low temperature (80 K) reflectivity spectra recorded on eight MBE grown Zn_1-x_Mn_x_Se/GaAs (001) epilayers with *x* ≤ 0.78. The spectrum of each material sample is shifted upwards on the reflectivity scale for clarity. We have achieved three major outcomes after analyzing the FIR spectral data on Zn_1-x_Mn_x_Se: (a) identified optical phonons of its constituent members, ZnSe and MnSe; (b) recognized a weaker mode of Mn-alloy disorder; and (c) established the classical ‘intermediate-phonon-mode’ behavior.

Depending upon the atomic masses of Zn, Mn and its bonding with Se atoms we have classified the distinctive optical phonons of ZnSe, MnSe in Zn_1-x_Mn_x_Se (epilayer) and GaAs (substrate). Different color arrows are used to designate the *ω*_*TO*_ modes of ZnSe, MnSe, and *ω*_*TO*_, *ω*_*LO*_ phonons of GaAs. In Figure 2, the reflectance spectrum for *x* = 0 (0.78) offered ZnSe (MnSe) like *ω*_*TO*_ mode frequency near 208.5 cm^−1^ (219.3 cm^−1^). Again, we have noticed *ω*_*TO*_ phonons of ZnSe (MnSe) and GaAs displaying blue shifts by ~3–4 cm^−1^ (~5 cm^−1^) and ~4 cm^−1^ when the temperature is decreased from 300 K to 80 K. For Zn_1-x_Mn_x_Se, the composition dependent optical modes and their broadening parameters – derived from theoretical fits to the experimental reflectivity spectra (cf. Section 4.2) are reported in Table 1.

For a sample with lower Mn-composition (*x* = 0.07) one can notice (see Table 1) nearly equal values of MnSe-like *ω*_*TO*_, 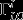 modes and their broadening parameters Γ_*TO*_, 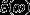 – suggesting that the dielectric function 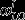 evaluated from the product rule [Equation (3)] becomes virtually identical to the one obtained from the sum rule [Equation (2)]. In samples with larger *x*, the difference between MnSe-like *ω*_*TO*_, 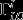 and Γ_TO_, 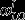 becomes significantly distinctive – accentuating the importance of Equation (3) for accurately modeling (cf. Section 4.2) the reflectivity spectra of alloys. Besides perceiving ZnSe- and MnSe-like *ω*_*TO*_ resonances (Figure 2), we have also noticed a weaker phonon feature below the MnSe-like *ω*_*TO*_ band – labeled by a dark blue color arrow. By increasing the Mn-composition (or decreasing temperature) – the weaker mode has revealed red (or blue) shift comparable to the ZnSe- and MnSe-like *ω*_*TO*_ resonances. This observation has eliminated the possibility of labeling a weaker mode to an experimental artifact. We have linked this phonon feature to Mn-alloy disorder instigated by increasing *x* – that led to the formation of clusters and/or complexes. In earlier measurements [60,61] on semiconductor alloys – the prospects of microscopic changes in the neighboring atomic environments have confirmed perceiving similar types of weak phonon-modes.

**Figure 3.**
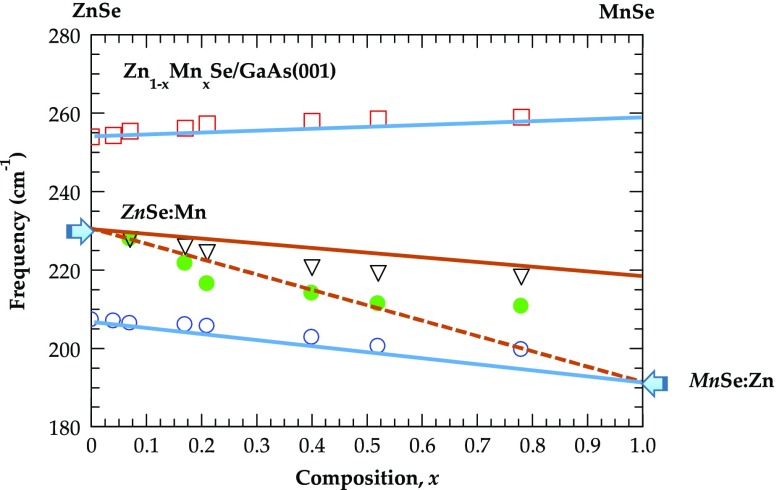
Composition dependent phonon frequencies of Zn_1‴*x*_Mn_*x*_Se ternary alloys. The symbols (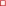 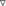 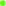 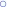) represent the phonon frequencies of (ZnSe-like LO), (ZnSe-like TO), (MnSe-like TO) and (MnSe-like LO^I^) modes. These frequencies were extracted from theoretical fits (see Table 1) to the FIR data (cf. Figure 2). Based on composition dependent frequencies, the Zn_1‴*x*_Mn_*x*_Se alloy exhibits an ‘intermediate phonon mode’ behavior. In this category, for *x* ~ 0 a triply degenerate Mn impurity mode in ZnSe (i.e. *Zn*Se:Mn) near ~230 cm^−1^ appears between ZnSe-like LO-TO (254 cm^−1^ -208 cm^−1^) phonons. As the composition *x* varies, the impurity mode splits up into MnSe-like TO (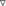) and MnSe-like longitudinal mode LO^I^ (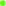). The later mode merges with ZnSe-like TO mode (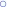) at the extreme limit *x* ~ 1 to create a triply degenerate Zn impurity mode in zb MnSe (i.e. *Mn*Se:Zn) near ~197 cm^−1^ which falls below the *zb* MnSe-like LO-TO phonons (258–219 cm^−1^) (see text).

Based on extended category of composition dependent optical phonons in ternary alloys our analysis of FIR spectra in Zn_1-x_Mn_x_Se suggested a classical ‘intermediate-phonon mode’ behavior in corroboration with others.[21–28] In this classification, the MnSe-like features fall within the ZnSe-like *ω*_*TO*_, *ω*_*LO*_ modes. At the lowest composition limit *x* ~ 0, one expects a triply degenerate Mn impurity mode (Figure 3) to appear near ~230 cm^−1^ between ZnSe *ω*_*TO*_ (≡ 208.5 cm^−1^), *ω*_*LO*_ (≡ 254.0 cm^−1^) phonons. As *x* increases the impurity mode splits up into MnSe-like *ω*_*TO*_ and an LO mode designated by 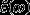
(dotted line) which in the extreme limit *x* ~1 merges with ZnSe-like *ω*_*TO*_ mode to create a triply degenerate Zn impurity mode near ~197 cm^−1^ below the zb MnSe *ω*_*TO*_ (≡ ~219.3 cm^−1^), *ω*_*LO*_ (≡ ~258.0 cm^−1^) phonons.

### 4.2. Modeling the reflectivity spectra at near normal incidence

**Figure 4.**
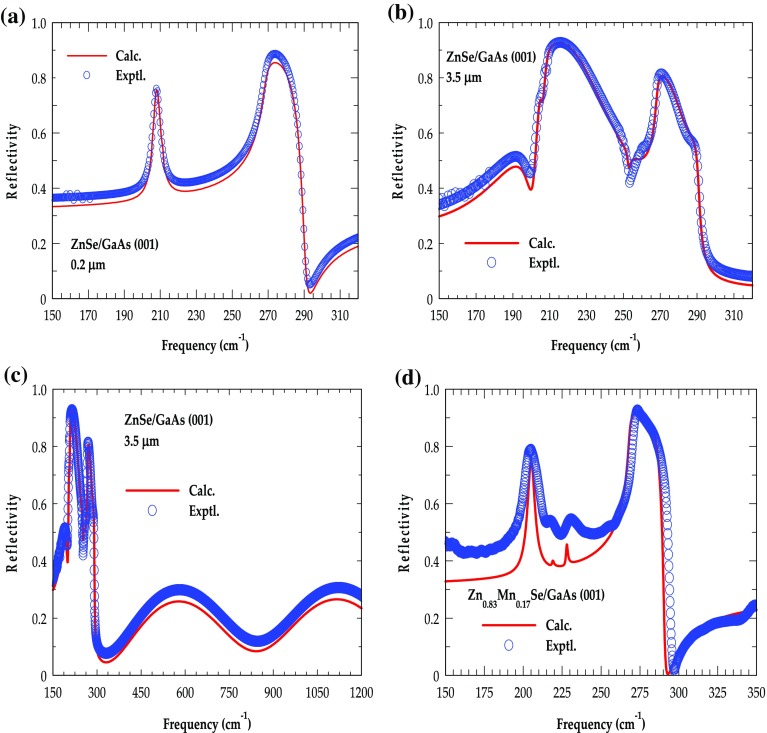
Comparison of simulated (red line) and experimental reflectance (blue open circles) at near normal incidence for (a) ZnSe/GaAs (001) epilayer of thickness 0.2 μm between 150 and 320 cm^−1^; (b) ZnSe/GaAs (001) of thickness 3.5 μm between 150 and 320 cm^−1^; (c) ZnSe/GaAs (001) of thickness 3.5 μm between 150 and 1200 cm^−1^; and (d) Zn_0.83_Mn_0.17_Se/GaAs (001) of thickness 0.2 μm between 150 and 350 cm^−1^ (see text).

By exploiting the classical theory (cf. Sections 3.1–3.2) of reflectivity, we have simulated (see Figure 4 (a–d)) the FIR spectra for a few undoped samples of Zn_1-x_Mn_x_Se/GaAs (001) with different epilayer thickness. The calculated reflectivity spectra shown by solid (red color) lines compare favorably well with the experimental data symbolized by (blue color) open circles. In the numerical calculations of 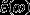 and/or reflectivity for the ZnSe/GaAs (001) epilayers, we have used *ω*_*TO*_ (≡ ~208.5 cm^−1^), *ω*_*LO*_ (≡ ~254 cm^−1^) and its broadening parameters Γ_*TO*_ = Γ_*LO*_ (≡ 1.7) listed in Table 1.

The reflectivity spectra (see Figure 4(a–c)) revealed significant changes in the shape of ZnSe and GaAs reststrahlen bands as the epilayer thickness is increased from 0.2 μm to 3.5 μm. By inspecting the spectra of 0.2 μm thick ZnSe/GaAs (001) epifilm, we find a sharp (narrow) peak near ZnSe *ω*_*TO*_ mode along with full reststrahlen band of GaAs (see Figure 4(a)) and no interference fringes. A thicker ZnSe/GaAs (001) epifilm can provide enough medium for the evanescent wave to propagate in ZnSe causing nearly full ZnSe- and GaAs-like reststrahlen bands to emerge (Figure 4(b)) along with interference fringes to appear at higher frequency (Figure 4(c)). For simulating the reflectivity spectra of Zn_0.83_Mn_0.17_Se/GaAs (001) epilayer (Figure 4(d)) – we find the best-fit model for evaluating lattice contributions to simulate 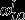 using Equation (3) required *m* = 3, where (cf. Table 1) *i* = 1 stands for ZnSe-like with *ω*_*TO*_ (≡ 206.1 cm^−1^), *ω*_*LO*_ (≡ 256.2 cm^−1^), Γ_*TO*_ (≡ 4.07), Γ_*LO*_ (≡ 4.13); *i* = 2 for MnSe-like with *ω*_*TO*_ (≡ 226.4 cm^−1^), 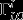 (≡ 221.7 cm^−1^), Γ_*TO*_ (≡ 9.32), 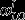 (≡ 7.27) and *i* = 3 for unknown-type (cluster?) with *ω*_*TO*_ (≡ 220.4 cm^−1^), 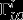 (≡ 219.9 cm^−1^), Γ_*TO*_ (≡ 7.92), 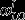 (≡ 8.39). The comparison of simulated reflectivity spectra with the experiment (cf. Figure 4(d) has supported our earlier assertion that Zn_1-x_Mn_x_Se exhibits a typical ‘intermediate phonon-mode’ behavior where the MnSe-like modes (*ω*_*TO*_, 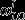) are located within the LO-TO phonons of ZnSe.

### 4.3. Reflectivity and transmission spectra of epilayers at oblique incidence

In the reflectivity and/or transmission studies of ultrathin polar epifilms it has been anticipated theoretically and verified experimentally [40] that in the FIR spectra at oblique incidence only *ω*_*TO*_ phonon is envisioned in the *s*-polarization while both *ω*_*TO*_ and *ω*_*LO*_ modes are expected in the *p*-polarization. Such an exposition for ultrathin Zn_1-x_Mn_x_Se/GaAs (001) epifilms and (MnSe)_m_/(ZnSe)_n_ SLs would be valuable in offering a strong basis for analyzing the reflectivity/transmission spectra and establishing the long wavelength optical phonons in the technologically important materials.

**Figure 5.**
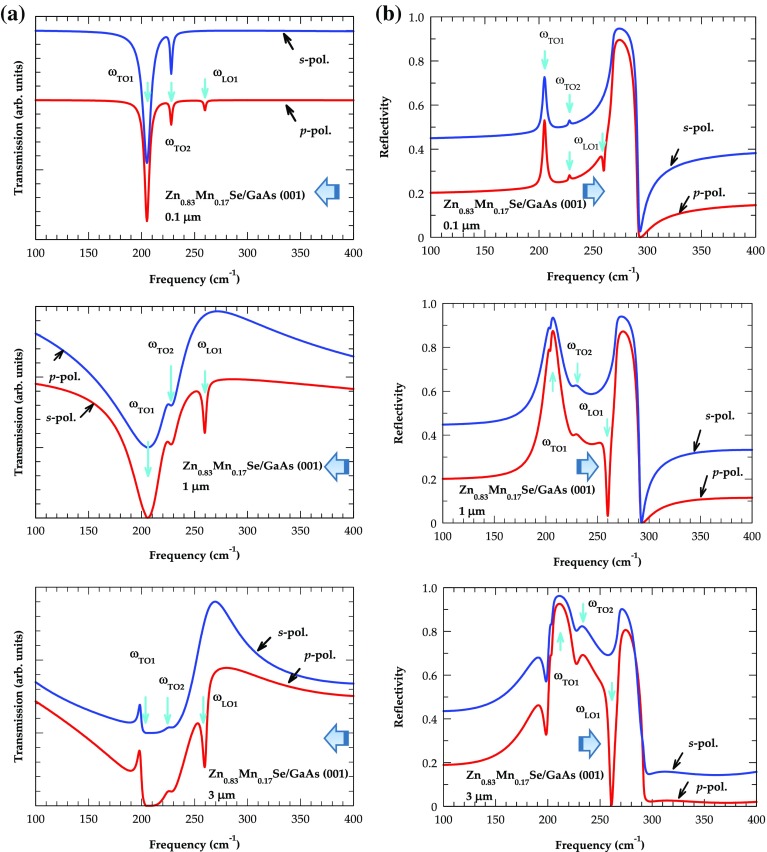
Thickness dependent simulated (a) transmission and (b) reflectance spectra of Zn_0.83_Mn_0.17_Se/GaAs (001) epilayers at oblique incidence (*θ*_*i*_ = 45^◦^). The *s*- (blue line) and *p*-polarization (red line) spectra are displayed with increasing thickness from 0.1 μm to 3.0 μm and corroborate the Berreman’s effect (see text).

In Figure 5(a) and (b) we report the results of simulated transmission (left panel) and reflectivity spectra (right panel) at oblique incidence (*θ*_i_ = 45^o^) for Zn_0.83_Mn_0.17_Se/GaAs (001) epilayers with different film thicknesses (0.1, 1.0 and 3.0 μm) following the procedures outlined in Sections 3.2–3.4 and incorporating the values of ZnSe-like (MnSe-like) phonons *ω*_*TO*_, *ω*_*LO*_ (*ω*_*TO*_, 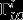) and broadening Γ_*TO*_, Γ_*LO*_ (Γ_*TO*_, 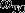) parameters from Table 1. Clearly, the transmission spectra (see Figure 5(a)) in the *s*-polarization (*T*_*s*_) revealed dips near the transverse optical mode of ZnSe marked as *ω*_*TO*1_ (≡ ~206 cm^−1^) and of MnSe denoted as *ω*_*TO*2_ (≡ ~222 cm^−1^), respectively. However, the transmission (*T*_*p*_) spectra in the *p*-polarization provided dips near *ω*_*TO*1_, *ω*_*TO*2_ and *ω*_*LO*1_ (≡~256 cm^−1^) – in good agreement with experimental data.[21–28] Again, our simulation results have demonstrated a clear progression of the two transmission dips for ultrathin film (0.1 μm) to a broad region of transmission covering nearly the entire reststrahlen band for relatively thicker (≥3.0 μm) films. Like transmission spectra, we have displayed results of the calculated reflectivity spectra (see Figure 5(b)) at oblique incidence (*θ*_i_ = 45^o^) for Zn_0.83_Mn_0.17_Se/GaAs (001) with epilayer thicknesses identical to the one used in Figure 5(a). It should be noted that in semiconductors the reflectance spectra (*R*_*s*_) generally drops to a minimum at the plasma edge which depends upon the carrier concentration to exhibit a peak near *ω*_*TO*_ mode. In semi-insulating materials with *ω*_*p*_ ≈ 0, the 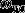 phonon peak, however, dominates at near-normal incidence while at oblique incidence (*θ*_i_ = 45^o^) a maxima is expected to occur at 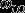 and a dip near ~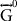 (Berreman’s effect [40]) in the *p*-polarization (*R*_*p*_) reflectivity (see Figure 5(b)).

### 4.4. Reflectivity and transmission of (ZnSe)_m_/(MnSe)_n_/GaAs (001) SLs

**Figure 6.**
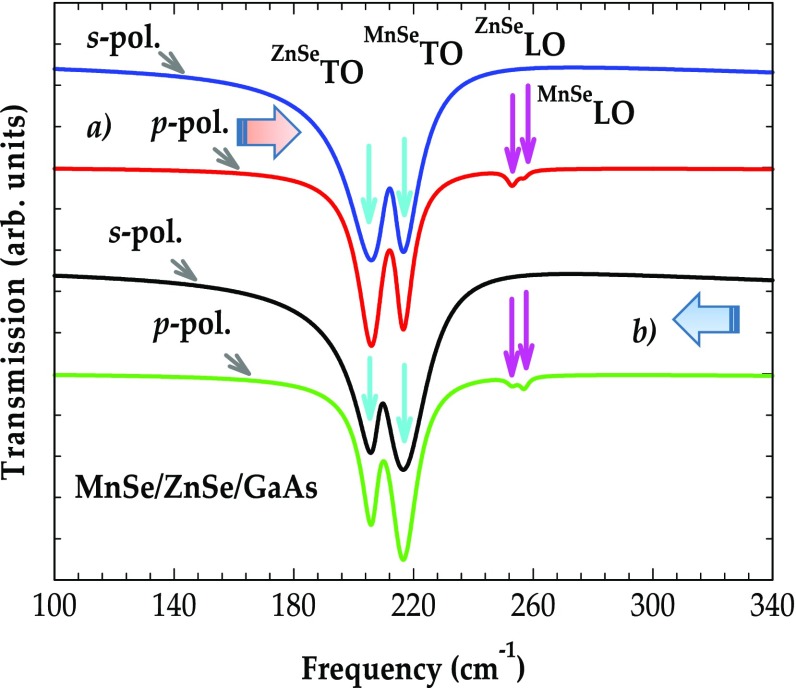
Simulated *s*- and *p*- polarization transmission spectra at oblique incidence (*θ*_i_ = 45^o^) as a function of frequency for: (a) 1 μm thick zb MnSe epilayer with 2 μm thick ZnSe buffer layer on GaAs (001) substrate – top two graphs; and (b) 2 μm thick zb MnSe epilayer with 1 μm thick ZnSe buffer layer on GaAs (001) substrate – bottom two graphs. The ZnSe-, MnSe-like TO modes are represented by light blue color arrows while their respective LO modes are shown by magenta color arrows (see text).

In Figure 6, we have displayed the results of simulated transmission spectra at oblique incidence (*θ*_i_ = 45^o^) for 1 μm (2 μm) thick zb MnSe epilayer with 2 μm (1 μm) thick ZnSe buffer layer prepared on GaAs (001) substrate. In the *s*-polarization transmission spectra, as expected, we have noticed minima occurring near *ω*_*TO*_ phonons of the MnSe epilayer (^MnSe^TO ~ 219 cm^−1^) and of the ZnSe buffer layer (^ZnSe^TO ~ 208 cm^−1^) while in the *p*-polarization spectra both *ω*_*TO*_ and *ω*_*LO*_ phonons (dips) of the MnSe (e.g. ^MnSe^TO ~ 219 cm^−1^, ^MnSe^LO ~ 258 cm^−1^) and of the ZnSe (^ZnSe^TO ~ 208 cm^−1^, ^ZnSe^LO ~254 cm^−1^) are revealed. It is to be noted that as the epilayer (buffer layer) thickness increases (decreases) from 1 μm → 2 μm (2 μm → 1 μm) the transmission dips show increase (decrease) near the ^MnSe^TO, ^MnSe^LO (^ZnSe^TO, ^ZnSe^LO) frequencies [see Figure 6(a) top two graphs and (b) bottom two graphs].

**Figure 7.**
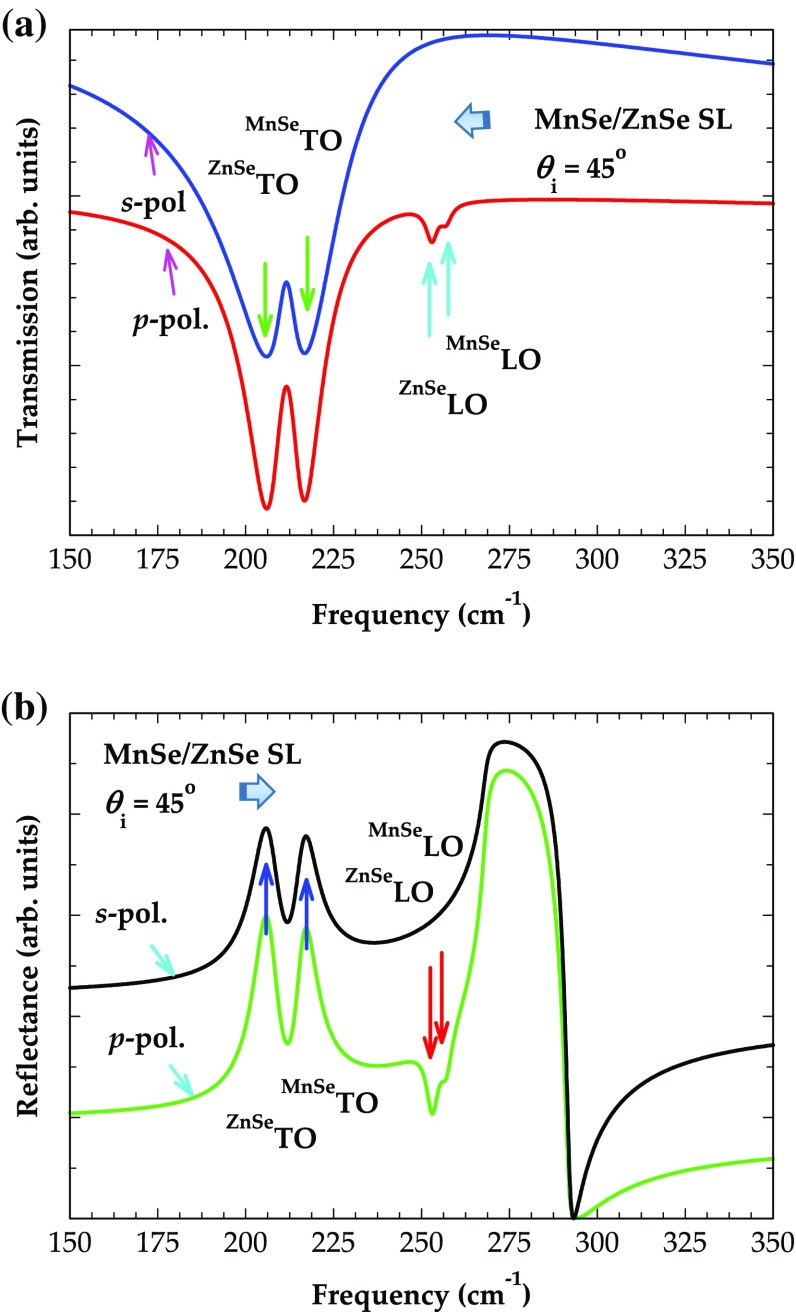
Simulated transmission and reflectance spectra at oblique (*θ*_i_ = 45^o^) incidence for MnSe/ZnSe SL on GaAs (001) as a function of frequency (cm^−1^): (a) transmission spectra; and (b) reflectance spectra. The SL consisted of 100 repeat periods of 30 Å MnSe and 20 Å ZnSe (see text).

Next we present our theoretical results of the transmission (see Figure 7(a)) and reflectivity (Figure 7(b)) spectra for (MnSe)_m_/(ZnSe)_n_/GaAs (001) SL – examining a structure consisting of 100 repeat periods of 30 Å thick zb-MnSe and 20 Å thick ZnSe layers on GaAs. Consistent with our earlier observations (cf. Figures 5 and 6) the simulations of the transmission spectra in the *s*-polarization reveal two minima (see Figure 7(a)) at the *ω*_*TO*_ modes of ZnSe, zb-MnSe layers and four minima in the *p*-polarization – two at *ω*_*TO*_ and two at *ω*_*LO*_ mode frequencies of ZnSe, zb-MnSe layers, respectively. In the reflectivity spectra, however, we noticed (see Figure 7(b) two maxima at ^ZnSe^TO, ^MnSe^TO in the *s*-polarization while in the *p*-polarization the study revealed two maxima at ^ZnSe^TO, ^MnSe^TO and two additional dips (one pronounced and another weak) at ^ZnSe^LO, ^MnSe^LO, respectively. One must also note that as the *ω*_*TO*_ and *ω*_*LO*_ modes in the ZnSe and zb-MnSe layers are separated by ~ 11 cm^−1^ and ~ 4 cm^−1^, respectively, the simulated results of the transmission and reflectance spectra providing optical phonons at suitable frequencies with apposite splitting has clearly elucidated the importance of the present methodology. In the absence of experimental results of the FIR reflectance and transmission spectra for (MnSe)_m_/(ZnSe)_n_/GaAs (001) SLs, our simulation has provided a compelling validation to the observed IR transmission data of CdSe/ZnTe SL grown on GaAs (001) [39].

### 4.5. Lattice dynamics of Zn-Mn chalcogenides

In zb semiconductor materials, the LVM spectroscopy plays an important role studying the structural and composition dependent phonon characteristics especially for identifying the site selectivity of defects. From a theoretical standpoint, one must note that the existing methods of calculating impurity modes in the disordered semiconductors are not sufficiently sophisticated to make accurate predictions of their vibrational traits. In the framework of an ATM-GF theory [50] it has now become possible to define reasonably accurate perturbation matrices by integrating the estimated radial force constant variations derived from the extended X-ray fine structure (EXAFS) measurements for comprehending the composition dependent phonon modes in zb ternary alloys.[50]

**Figure 8.**
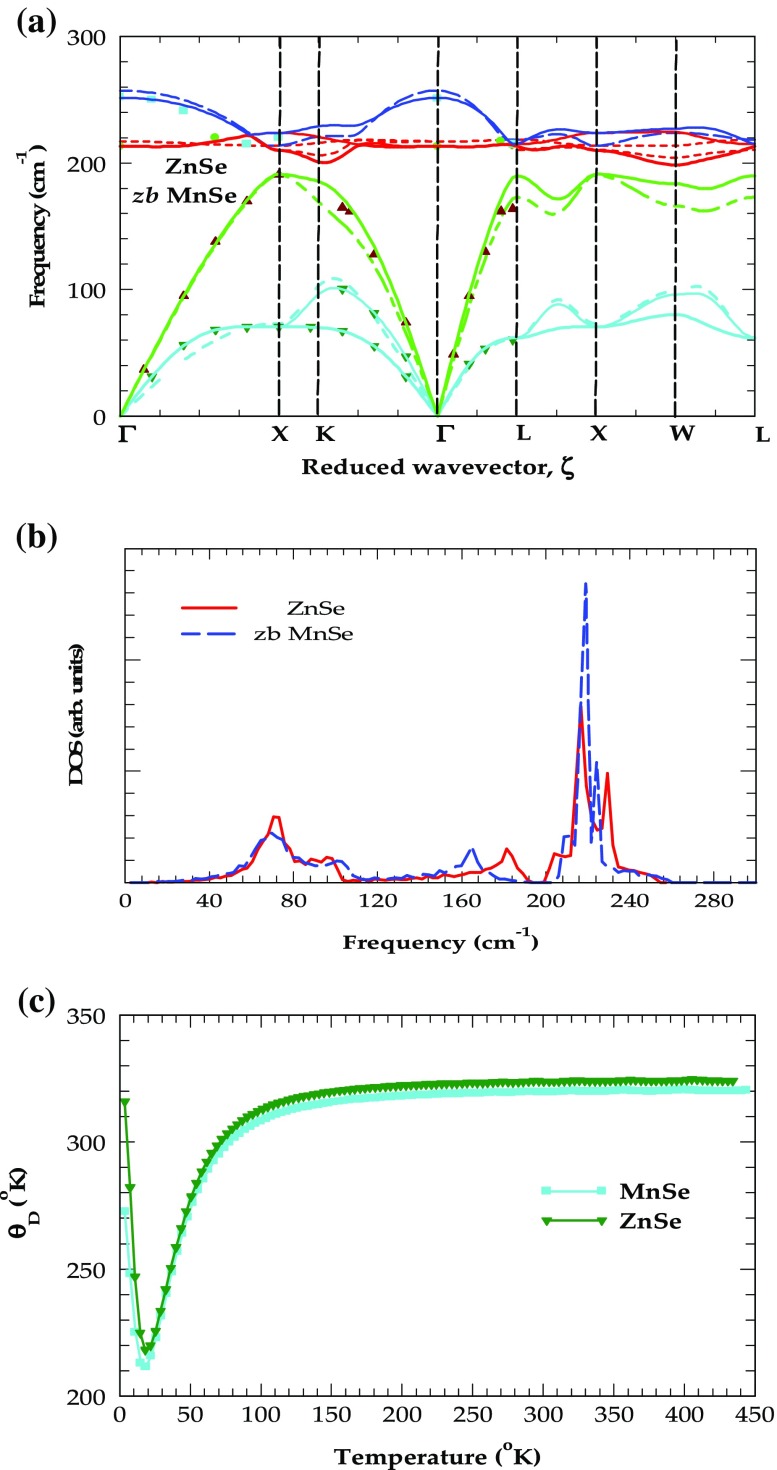
(a) The results of an optimized rigid-ion-model calculations for phonon-dispersions along high-symmetry directions:[52] ZnSe (solid lines) are compared with inelastic neutron scattering data (symbols),[43] zb MnSe (dotted lines); (b) simulated one-phonon density of states for ZnSe (solid lines) and zb MnSe (dotted lines); (c) simulated Debye temperatures for ZnSe (solid lines) and zb MnSe (dotted lines).

To recognize the impurity vibrational modes of p-doped Zn_1−x_Mn_x_Se alloys within the ATM-GF framework, one needs to evaluate the elements of the lattice Green’s function 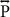
and perturbation 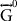 matrices by incorporating the host lattice phonons (i.e. eigenvalues and eigenvectors) from a reliable lattice dynamical scheme.[50] Before probing the composition dependent LVMs of N and P impurities in Zn_1−x_Mn_x_Se we report in Figure 8(a), (b) and (c) the simulated phonon dispersions, density of states (DOS) and Debye temperatures of ZnSe and zb-MnSe derived from an optimized rigid-ion-model [58] fitted to the inelastic neutron scattering [46–48] and first-principle data.[49] The RIM values of phonon energies at high critical-points and the values of Debye temperature [Θ_D_(min) and Θ_D_(T → 0)] reported in Table 2 are shown to possess the required accuracies by comparing them with the existing experimental data.[46–48]

**Table 2.** Rigid-ion model calculations of phonon frequencies (in wave numbers) at high critical points in the Brillouin zone, Debye temperatures (in °K) are compared with the existing experimental and theoretical data for ZnSe, and MnSe.

Critical points	ZnSe [experimental]	ZnSe [this study]	MnSe [other studies]	MnSe [this study]
LO(Γ)	252^a^	251.6	258.0^c^	257.2
TO(Γ)	213^a^	213.1	218.0^c^	217.2
LO(X)	210^a^	209.8	–	210.7
TO(X)	220^a^	223.8	–	213.7
LA(X)	191^a^	191.1	–	190.0
TA(X)	70^a^	70.4	–	73.1
LO(L)	215^a^	213.1	–	211.2
TO(L)	214^a^	214.9		218.3
LA(L)	164^a^	189.9		171.6
TA(L)	60^a^	61.9		61.4
Θ_D_(min)	210.0^b^	218.0		211.5
Θ_D_(T→0)	339^b^	305.7		272.7

^a^ [46],

^b^ [65],

^c^ [22].

### 4.6. Impurity vibrational modes

**Figure 9.**
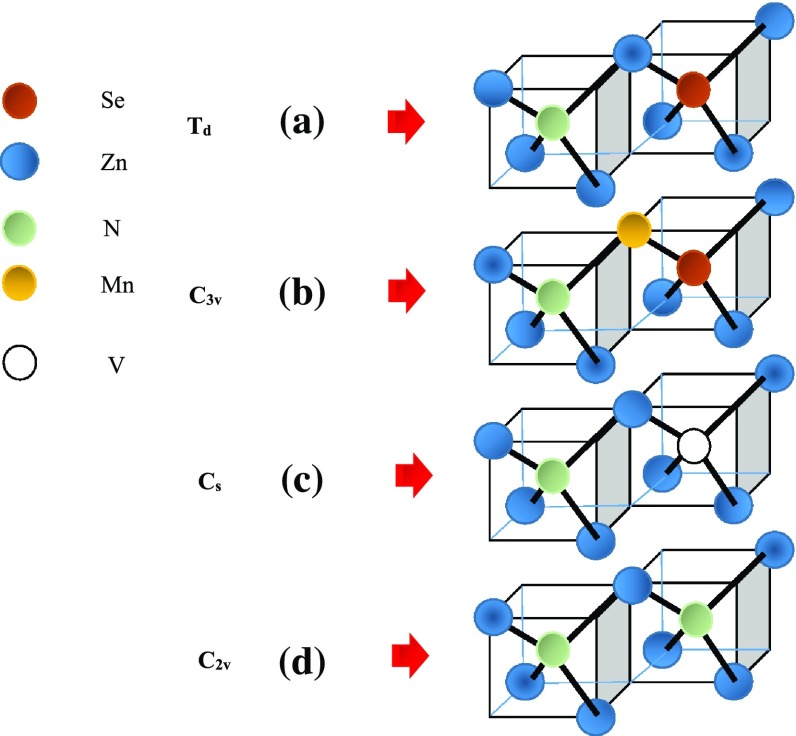
Sketches of the perturbation models for various defects in zb ZnSe: (a) an isolated defect say N-occupying Se-site (i.e. N_Se_) in ZnSe (*T*_d_ symmetry); (b) nearest-neighbor pair say N-defect occupying Se-site and paired with Mn-defect occupying Zn-site (i.e. N_Se_-Mn_Zn_) in ZnSe (*C*_3v_ symmetry); (c) second-nearest neighbor complex involving two-different defects say N-occupying Se-site and paired with vacancy on Se-site (i.e. N_Se_-Zn-V_Se_) in ZnSe (*C*_s_ symmetry); and (d) second nearest neighbor complex involving two-identical defects say N-occupying Se-site and paired with N- on Se-site (i.e. N_Se_-Zn-N_Se_) in ZnSe (*C*_2v_ symmetry).

In Zn_1−x_Mn_x_Se doped with N and P impurities, we have investigated the impurity vibrational modes of various isolated (*T*_d_), NN pair (*C*_3v_) and 2nd NN complex (*C*_s_/*C*_2v_) defect centers (cf. Figure 9(a–d)) by integrating the RIM phonons to calculate the Green’s function and perturbation matrices. In the framework of an ATM-GF theory and using the appropriate 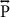 and 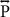 matrices in different defect configurations [50] the simulated LVM frequencies are compared and discussed with the existing IR and RS data.[51, 52] Here we report the outcomes of impurity vibrational modes for the following cases:

(a) Single isolated defects

**Figure 10.**
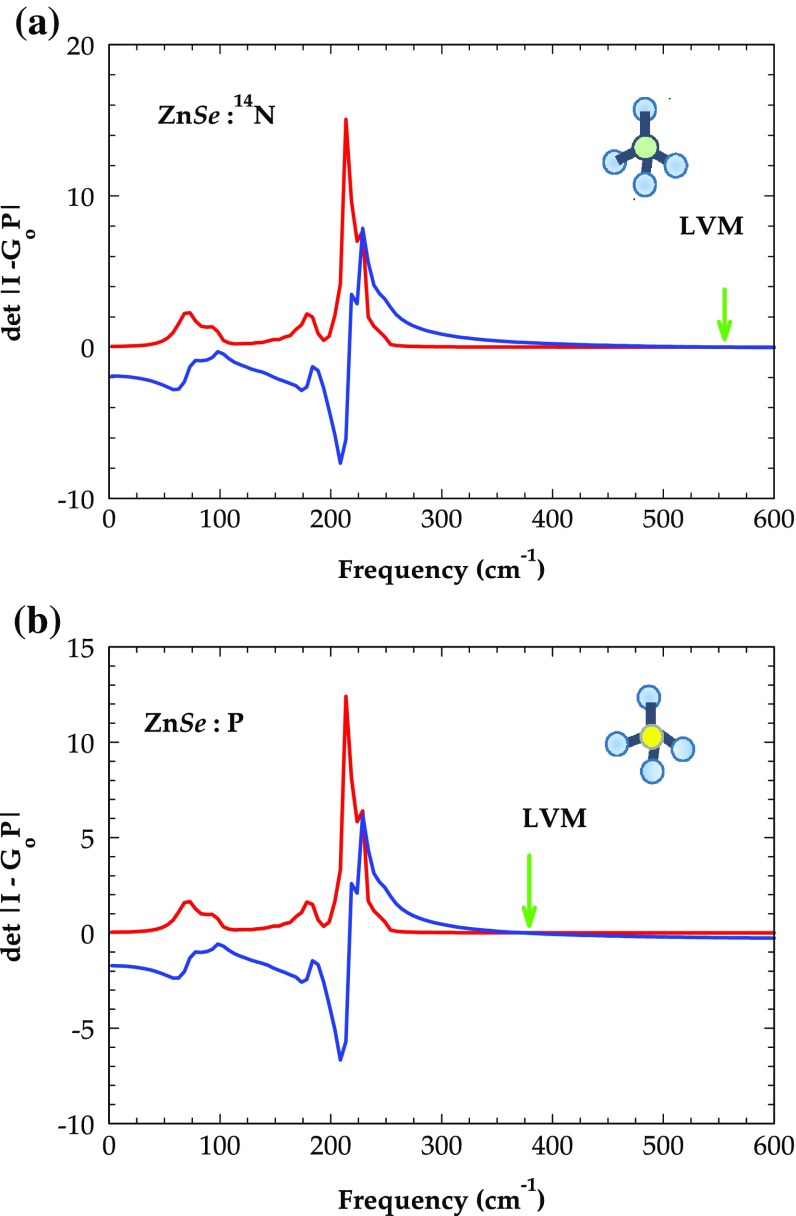
Calculated real (blue) and imaginary (red line) parts of the det | *I* − *G* ^o^ *P* | (cf. Section 3.5) in the *F*_2_ representation. The crossing of real part of det | *I* − *G* ^o^ *P* | in the region having zero density of phonon states provides local mode of (a) ^14^N_Se_ in Zn*Se*, and (b) P_Se_ in Zn*Se*.

In lightly doped p-ZnSe (p-MnSe) we first estimated the impurity–host interactions for isolated ^14^N_Se_ (^15^N_Se_) and P_Se_ defects (*T*_d_ – symmetry) by examining the effects of lattice relaxation on the force constants by using Harrison’s semi-empirical bond-orbital model (BOM).[62] In terms of the Hartree–Fock atomic term values, the BOM provided simple analytical expressions for the change in the impurity-host (NN) and host-host (2nd NN) bond energies [63] and suggested a computationally efficient and adequate method to appraise the bond-length distortions. Next we built the full size (15 × 15) 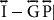 matrix in the framework of RIM following the method described elsewhere.[45] The simulated results for the real (full lines) and imaginary (dashed lines) parts of the det |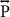 in triply degenerate *F*_2_ irreducible representation of *T*_d_ symmetry [cf. Equation (10)] are displayed in Figure 10(a) and (b) for Zn*Se*:^14^N and Zn*Se*:P, respectively. The crossing of real part of the determinant through zero above the maximum (*ω*_m_~ 254 cm^−1^) phonon frequency of ZnSe has provided LVMs for ^14^N_Se_ near ~552 cm^−1^ and for P_Se near_ ~374 cm^−1^, offering a strong corroboration to the observed IR [51] and Raman scattering spectroscopy [52] data. Similar results of the LVMs for ^14^N_Se_ (^15^N_Se_) and P_Se_ defects in zb-MnSe are included in Table 3.

**Table 3.** Comparison of the calculated triply degenerate *F*_2_ localized-vibrational modes by ATM-Green’s function theory with experimental data for various isolated low-mass defects (*T*_*d*_-symmetry) in ZnSe and MnSe.

System	Local vibrational modes (cm^−1^)		
	Calculated^a^ (by including ‘impurity-host’ interaction)	Experimental^b^	Force constant variation
*Zn*Se:^6^Li	350.00	350.0	0.520
*Zn*Se:^7^Li	329.00	329.0	0.520
*Zn*Se:Be	450.00	450.0	−0.040
*Zn*Se:^24^Mg	305.00	305.0	−0.080
*Zn*Se:^25^Mg	300.70	–	−0.080
*Zn*Se:^26^Mg	296.5	–	−0.080
*Zn*Se:Al	359.00	359.0	−0.600
Zn*Se*:^14^N	552.70	553.0	−0.630
Zn*Se*:^15^N	536.00	537.0	−0.630
Zn*Se*:^32^S	297.00	297.0	0.160
Zn*Se*:^33^S	293.06	–	0.160
Zn*Se*:^34^S	289.30	–	0.160
Zn*Se*:P	374.70	375.0	−0.500
Mn*Se*:^14^N	518.30	–	−0.62
Mn*Se*:^15^N	503.20	–	−0.62
Mn*Se*:P	358	–	−0.50

^a^ Present theory.

^b^ [45] and references cited therein.

(b) Nearest neighbor pair defects

For the low concentration of p-type {e.g. N (P)} dopants in dilute Zn_1−x_Mn_x_Se (*x* < 0.03) alloys, one expects splitting of the triply degenerate (*F*_2_) LVM of Zn*Se*:^14^N (Zn*Se*:P) near ~552 cm^−1^ (near ~374 cm^−1^) due to Mn alloying. If one pragmatically assumes the statistical distributions of Mn atoms occupying the Zn sites in Zn_1−x_Mn_x_Se, there is only one probable arrangement that establishes the formation of NN pairs of *C*_3v_ symmetry (cf. Figure 9(b)) involving Mn_Zn_ and ^14^N_Se_ (P_Se_), i.e. Mn_Zn_-^14^N_Se_ (Mn_Zn_-P_Se_). For such a pair defect including eight atoms with axis along the pair-bond the defect space would increase [45] from 15 × 15 to 24 × 24. From group-theoretical analysis, both *A*_1_ and *E* types of modes for *C*_3v_ symmetry are perceived to be optically active. As the degeneracy at the N_Se_ (P_Se_) site is lifted due to Mn alloying, the ATM-GF calculation [50] for Mn_Zn_-^14^N_Se_ (Mn_Zn_-P_Se_) pairs ascribed two modes (see Table 4), one *A*_1_ appearing at a lower frequency and the other *E* at a higher frequency of the main ^14^N_Se_ (P_Se_) *F*_2_ local mode. In the absence of experimental results for p-doped Zn_1−x_Mn_x_Se, our simulations offered compelling validations to impurity modes by IR spectroscopy [64] of low phosphorous dopant levels in dilute Zn_1−x_Mn_x_Te (*x* < 0.025).

**Table 4.** Comparison of calculated localized-vibrational modes (LVMs) for the nearest-neighbor and next-nearest neighbor defects in lightly and heavily doped N and P in Zn_1−x_Mn_x_Se (*x* < 0.03) by using an ATM-Green’s function theory.

Local vibrational modes (cm^–1^)			
System	Symmetry	Calculated^a^	Force constant variations ^a^
			*t* = *F*_12_; *u* → C_3v_
			*t* = *F*_12_; *u = F*_26_*; v* → C_s_ (C_2v_)
*Lightly doped N and P in Zn*_*1−x*_*Mn*_*x*_*Se*			
Mn_Zn_-^14^N_Se_	*C*_3v_	554.98 (*E*)	0.03, –0.63
		549.60 (*A*_1_)	

Mn_Zn_-^15^N_Se_	*C*_3v_	537.48 (*E*)	0.03, –0.63
		532.40 (*A*_1_)	

Mn_Zn_-P_Se_	*C*_3v_	375.98 (*E*)	0.03, –0.50
		373.72 (*A*_1_)	
*Heavily doped N and P in ZnSe and Zn*_*1−x*_*Mn*_*x*_*Se*			
^V^_Se_-Zn-^14^N_Se_	*C*_s_	557.2 (*A*_1_)	1.00, 0.0, –0.63
		552.4 (*A*_1_)	
		550.1 (*A*_2_)	

^V^_Se_-Zn-^15^N_Se_	*C*_s_	539.9 (*A*_1_)	1.00, 0.0, –0.63
		535.1 (*A*_1_)	
		532.6 (*A*_2_)	

^V^_Se_-Zn-P_Se_	*C*_s_	378.3 (*A*_1_)	1.00, 0.0, –0.50
		374.2 (*A*_1_)	
		372.7 (*A*_2_)	

^14^N_Se_-Zn-^14^N_Se_	*C*_2v_	573.51 (*A*_1_)	–0.63, 0.0, –0.63
		568.72 (*B*_1_)	
		553.20 (*B*_2_)	
		548.01 (*B*_1_)	
		547.22 (*A*_1_)	
		545.30 (*B*_2_)	

^15^N_Se_-Zn-^15^N_Se_	*C*_2v_	556.24 (*A*_1_)	–0.63, 0.0, –0.63
		550.07 (*B*_1_)	
		536.20 (*B*_2_)	
		531.47 (*B*_1_)	
		530.02 (*A*_1_)	
		529.60 (*B*_2_)	

^14^N_Se_-Mn_Zn_-^14^N_Se_	*C*_2v_	577.37 (*A*_1_)	–0.63, 0.03, –0.63
		569.67 (*B*_1_)	
		553.25 (*B*_2_)	
		548.03 (*B*_1_)	
		546.84 (*A*_1_)	
		545.45 (*B*_2_)	

^15^N_Se_-Mn_Zn_-^15^N_Se_	*C*_2v_	560.64 (*A*_1_)	–0.63, 0.03, –0.63
		551.67 (*B*_1_)	
		536.22 (*B*_2_)	
		532.07 (*B*_1_)	
		530.04 (*A*_1_)	
		529.80 (*B*_2_)	


^a^ [45].

(c) Second nearest neighbor complexes

In heavily doped p-ZnSe, it is interesting that as the concentration of N dopants exceed ~10^18^ cm^−3^ the material becomes highly compensated.[33–38] Extended total energy calculations have strongly contended that this kind of behavior could arise from self-compensation by ‘donor-acceptor’ pairs involving V_Se_ and N_Se_.[33–38] Again, the experimental data by positron annihilation, PL, and ODMR studies revealed two distinct N-related impurity levels – a shallow level is assigned to an isolated N_Se_ while a deep level is ascribed to the ‘defect-complex’ involving V_Se_ and N-impurity.[33–38] To comprehend the LVMs of the luminescent centers V_Se_-Zn-N_Se_ with *C*_s_ symmetry (cf. Figure 9(c)) or 2nd NN N-pairs of *C*_2v_ symmetry (cf. Figure 9(d)) we have constructed the complete representations of Γ_*C*s_ or Γ_*C*2v_ point-groups in the 33-dimensional space to block-diagonalize the 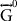 and 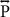 matrices.[45] From the group-theoretic arguments, it has been perceived that *A*_1_ and *A*_2_ (*A*_1_, *B*_1_, and *B*_2_) types of modes are optically active in the *C*_s_ (*C*_2v_) symmetry.

If the force constant change of an isolated ^14^N_Se_ (^15^N_Se_) defect (cf. Table 3) is retained in the prominent luminescence center ^V^_Se_-Zn-^14^N_Se_ (^V^_Se_-Zn-^15^N_Se_), our ATM-GF method [45] has predicted three non-degenerate impurity modes (see Table 4) near ~557.2, 552.4 and 550.1 cm^−1^ (~539.9, 535.1 and 532.6 cm^−1^) and their frequencies spanned within ~7 cm^−1^ – with similar calculations reported in Table 4 for dilute Zn_1−x_Mn_x_Se. For the 2nd NN light N-pair-defects N_Se_-Zn-N_Se_ (N_Se_-Mn_Zn_-N_Se_) in ZnSe (Zn_1−x_Mn_x_Se) with the *C*_2v_ symmetry our ATM-GF study has projected six impurity modes (see Table 4). By comparing the results of GF simulations with the existing room temperature IR spectra [51] for highly doped (~5 × 10^19^ cm^−3^) ^14^N and ^15^N in ZnSe – one discerns two broad absorption bands near ~553 cm^−1^ and 537 cm^−1^ having full width at half maximum (FWHM) of ~15 cm^−1^. Based on the ratio 1.030 of impurity modes for ^15^N to ^14^N with the segment 1.029 of reduced mass for diatomic molecule of N bonded to Zn, one study [51] validated the LVMs near ~553 cm^−1^ and 537 cm^−1^ to isolated substitutional N-isotopes occupying Se-sites. However, the effects of high concentration of N dopants on the perceived large absorption band widths of LVMs were not fully discussed.[51] As the concentration of N-dopants in ZnSe exceeds the effective acceptor activation limit, N-pairing is expected as assessed in many electrical measurements.[33–38] Unlike the LVMs of N-pair in Si, no such impurity modes are identified in ZnSe.[51] In elemental semiconductor Si, while the substitutional N_Si_ defects form pairs (*C*_3v_ symmetry) on identical nearest neighbor (NN) sites, the pairing of N in ZnSe, however, occurs on the adjacent anion sites (*C*_2v_ symmetry: Figure 9(d)). Moreover, the frequencies of six simulated LVMs for N_Se_-Zn-N_Se_ pair (see Table 4) falls within the range of nearly ~28–30 cm^−1^ – larger than the observed FWHM ~15 cm^−1^ of the isolated N_Se_ absorption band. We thus strongly rule out the possibility of N-N pairing in heavily doped p-ZnSe and endorse the formation of V_Se_-Zn-N_Se_ centers – evocative for the larger bandwidth observed in IR measurements.[51]

## 5. Summary and conclusions

In conclusion, we have presented the results of a comprehensive lattice dynamical study to emphasize the vibrational properties of perfect/imperfect zinc-blende (zb) binary ZnSe, MnSe, and ternary Zn_1−x_Mn_x_Se alloys. Low-temperature FIR reflectance spectra on a series of high quality MBE grown Zn_1−x_Mn_x_Se/GaAs (001) samples with large Mn composition range (*x* ≤ 0.78) has established the classical ‘intermediate-phonon-mode’ behavior. Besides observing ZnSe-like and MnSe-like *ω*_*TO*_ phonon resonances, we also noticed a weak phonon feature below the MnSe *ω*_*TO*_ band. By increasing Mn-composition (or decreasing temperature) the weak mode exhibited a red (or blue) shift similar to ZnSe- and MnSe-like *ω*_*TO*_ resonances. This observation has eliminated the possibility of relating the weak phonon trait to an experimental artifact and we have allied this feature to an alloy disorder mode.[60,61] Again, the rationale behind our FIR measurements on ultrathin MBE grown Zn_1-x_Mn_x_Se /GaAs (001) epilayers is to assess the validity of conventional effective-medium theory by comparing the simulated reflectivity spectra with experimental data. In the framework of a realistic RIM and exploiting an ATM-GF theory we have appraised the vibrational characteristics of nitrogen and phosphorous doped Zn-Mn chalcogenides. Lattice relaxations around N_Se_ and P_Se_ in ZnSe (zb MnSe) are estimated by using a first-principles BOM [62] to help construct 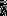 matrices for studying the LVMs. Consistent with the IR data [51] the ATM-GF simulation of impurity modes for isotopic ^14^N_Se_ (^15^N_Se_) defects in ZnSe offered strong revelation of inflexible impurity–host interactions. By retaining the force constant change parameter of isolated ^14^N_Se_ (^15^N_Se_) defects in one of the prominent PL centers ^V^_Se_-Zn-^14^N_Se_ (^V^_Se_-Zn-^15^N_Se_), our study has predicted three non-degenerate LVMs near ~557.2, 552.4, 550.1 cm^−1^ (~539.9, 535.1, 532.6 cm^−1^) with frequencies spanning to ~7 cm^−1^ and falling well within the FWHM (~15 cm^−1^) of ^14^N_Se_ absorption band.[51] A 2nd NN nitrogen pair defect N_Se_-Zn-N_Se_ of *C*_2v_ symmetry in ZnSe ensued six LVMs – covering a larger (~28–30 cm^−1^) range. In highly N-doped ZnSe, this assertion has ruled out the prospects of N-pairs and justified the presence of V_Se_-Zn-N_Se_ centers – likely to be responsible for the observed larger absorption bandwidth.[51] High resolution FIR and/or RS measurements are needed to validate the accuracy of our theoretical conjectures.

## Disclosure statement

No potential conflict of interest was reported by the authors.

## Funding

D.N.T. acknowledges an Innovation Research Grant that he received from the School of Graduate Studies at Indiana University of Pennsylvania, Indiana, PA, USA. T.R.Y. at the National Taiwan Normal University was supported by [grants number NSC 100-3113-S-003-004 and number NSC 100-3113-S-003-015].
